# Rapid analysis of formic acid, acetic acid, and furfural in pretreated wheat straw hydrolysates and ethanol in a bioethanol fermentation using atmospheric pressure chemical ionisation mass spectrometry

**DOI:** 10.1186/1754-6834-4-28

**Published:** 2011-09-06

**Authors:** Scott M Davies, Rob S Linforth, Stuart J Wilkinson, Katherine A Smart, David J Cook

**Affiliations:** 1 University of Nottingham, Division of Food Sciences, School of Biosciences, Sutton Bonington Campus, College Road, Loughborough, LE12 5RD, UK

## Abstract

Atmospheric pressure chemical ionisation mass spectrometry (APCI-MS) offers advantages as a rapid analytical technique for the quantification of three biomass degradation products (acetic acid, formic acid and furfural) within pretreated wheat straw hydrolysates and the analysis of ethanol during fermentation. The data we obtained using APCI-MS correlated significantly with high-performance liquid chromatography analysis whilst offering the analyst minimal sample preparation and faster sample throughput.

## Background

Conversion of lignocellulosic biomass into bioethanol currently involves four main processes: physicochemical pretreatment, enzymatic saccharification, fermentation and distillation and/or purification of ethanol. Pretreatment of lignocellulosic biomass is necessary to disrupt the inherently recalcitrant plant cell wall structure to facilitate enzyme access and thus hydrolysis of constituent polysaccharides to sugars in preparation for fermentation.

Current pretreatment processes employ high temperatures and/or extreme pH levels to disrupt and facilitate the separation of hemicellulose and lignin components of the cell wall [1]. However, owing to the nonspecificity of physicochemical pretreatments, a range of degradation products are formed, some of which are known to inhibit enzymatic saccharification and fermentation [2]. Such degradation products include weak organic acids, furans and phenolic compounds, all of which are known to independently and synergistically disrupt yeast metabolism, impairing fermentation and consequently limiting bioethanol yield if their concentrations are too high [2,3]. High levels of weak organic acids lead to acidification of the yeast cytosol. This cytosolic acidification depletes the intracellular ATP pool through a diversion of ATP to plasma membrane ATPases, which pump out H^+ ^ions to regulate the intracellular pH [2]. Furans are believed to inhibit key fermentative enzymes such as alcohol dehydrogenase, pyruvate dehydrogenase and aldehyde dehydrogenase [2]. Acetic acid in biomass hydrolysates is derived primarily from deacetylation of the hemicellulose xylan side chains. Formic acid meanwhile arises from the degradation of furfural or 5-hydroxymethylfurfural (HMF), both of which are derived from the dehydration of xylose or glucose, respectively [2,3]. Acetic acid and formic acid are reported to inhibit *Saccharomyces cerevisiae *at concentrations of 1.6 and 1.4 g/L, respectively [2].

Autohydrolytic pretreatment processes using water at temperatures up to 200°C have been shown to create acetic acid and formic acid [4]. Generation of these degradation products within the hydrolysate is unavoidable and necessary for the catalytic mechanism by which hemicellulose is hydrolysed from the cell wall, a process which is known to enhance enzymatic saccharification of the pretreated biomass [5,6].

Quantifying and screening pretreatment hydrolysates for degradation products and the analysis of ethanol in fermentation media are essential to assessing the efficacy of lignocellulosic biomass conversion into bioethanol. Analytical protocols for these analytes are typically based on gas chromatography (GC) or high-performance liquid chromatography (HPLC), both of which may involve long sample run times and often require extensive sample preparation prior to analysis. To overcome these limitations and address the requirement for high-throughput screening techniques [7], rapid analytical techniques such as full equilibrium headspace GC have been developed to identify furfural and ethanol within complex sample matrices [8,9]. As an alternative to these methods, we evaluated the suitability of atmospheric pressure chemical ionisation mass spectrometry (APCI-MS) as a rapid analytical tool for the analysis of key pretreatment degradation products (acetic acid, formic acid and furfural) and for the analysis of ethanol in samples taken from bioethanol fermentation. The rationale for using this technique extends from the research of Ashraf *et al. *[10], who used APCI-MS for the rapid analysis of aroma compounds in the headspace above alcoholic beverages. Their work showed that selected ion monitoring could offer selectivity in the analysis of aroma compounds, despite the lack of a chromatographic separation, and could be used to monitor their gas phase concentrations quantitatively.

In this study, we investigated the effects of sample headspace equilibration time, the impact of sample pH on headspace equilibration and the applied APCI-MS cone voltage were also explored and optimised. Cone voltage refers to the low voltage bias applied to the sampling cone in the APCI-MS source during analysis. Varying this parameter changes the fragmentation energy imparted to ions.

## Methods

### Chemicals

All chemicals used were of analytical grade (> 95% purity; Fisher Scientific, Loughborough, UK). Standard solutions of acetic acid, formic acid, furfural and ethanol were prepared by dispersing analytical grade reagents in deionised water to a final volume of 100 mL. Serial dilutions were then performed in deionised water to yield an appropriate range of calibration standards for analysis.

### Sample preparation

Wheat straw (cv. Zebedee) was harvested from the Sutton Bonnington University farm (University of Nottingham, Nottingham, UK) and milled using a knife mill fitted with a 3-mm screen (Fritsch, Idar-Oberstein, Germany).

#### Autohydrolytic pretreatment of wheat straw

Milled wheat straw (5 g) was transferred into a custom made stainless steel (316 grade) reactor vessel with end caps fitted with ferrules (Swagelok, Manchester, UK) and filled with 40 mL of deionised water to provide a 1:8 solid-to-liquid ratio.

To study the effects of pretreatment on wheat straw and to generate a range of inhibitor concentrations, a D-optimal designed experiment was created using Design-Expert version 7 software (Stat-Ease inc, Minneapolis, MN, USA). Pretreatment incubation temperatures (160°C, 180°C and 200°C) and residence times (10, 35 and 60 minutes) were varied to produce 18 experimental design points with duplicates at the extremities and centre of the design space. The reaction vessel was placed inside a Fisons GC 8000 Gas Chromatography oven (Fisons Instruments, Manchester, UK) set to the experimental pretreatment temperature and heated isothermally for the desired residence time. Following this step, the reaction vessel was placed into cold water to end the pretreatment process.

#### Bioethanol fermentation conditions

A small-scale (1.5 L) bioethanol fermentation was conducted using a synthetic medium comprising yeast, water, sugar (150 g/L glucose and 30 g/L xylose), yeast extract (10 g/L) and peptone (20 g/L). The fermentation was performed at 25°C, and samples (10 mL) were removed for analysis at time intervals of 0, 4, 8, 12, 23, and 27 hours to create samples with a range of ethanol concentrations for headspace analysis.

### High-performance liquid chromatography

Prior to HPLC analysis, all samples and standards were filtered using Whatman GD/X syringe filters (GF/C 25 mm filter diameter/1.2 μm pore size; Whatman International Ltd., Banbury, UK) and left undiluted. Furfural was analysed using a Waters 2695 HPLC system fitted with (1) a Waters 996 Photodiode Array Detector set (Waters Corp., Milford, MA, USA) to scan wavelengths between 230 and 280 nm and (2) a Techsphere ODS C18 column (5 μm, 4.6 mm × 250 mm; HPLC Technology, Macclesfield, UK). Separations were performed at ambient temperature. The mobile phase was a binary mixture of 1% acetic acid (solvent A) and methanol (solvent B) with an overall flow rate of 1.0 mL/minute. The system was operated in gradient mode, ramping from 20% to 50% methanol over 30 minutes with a 100% methanol column cleaning phase and a 9-minute reequilibration period. The sample injection volume was 10 μL. Analysis was completed within 45 minutes, and data were recorded using Waters Millennium Chromatography software (Waters Corp., Milford, MA, USA).

Acetic acid, formic acid and ethanol were analysed using an HPLC system composed of a JASCO AS-2055 Intelligent Autosampler (JASCO, Essex, UK), and a JASCO PU-1580 Intelligent HPLC Pump. Acetic acid and formic acid were separated on a Rezex ROA Organic Acid H^+ ^organic acid column (5 μm, 7.8 mm × 300 mm; Phenomenex, Macclesfield, UK) operated at ambient temperature with a 0.005 N H_2_SO_4 _mobile phase flowing at 0.5 mL/minute and detected using an LDC/Milton Roy Spectro Monitor 3000 variable wavelength UV detector (VG Laboratory Systems, Cheshire, UK) set to 210 nm. Sample analysis was completed in 27 minutes.

Ethanol was separated on a Rezex RPM Pb^2+ ^monosaccharide column (5 μm, 7.8 mm × 300 mm; Phenomenex) heated to 75°C using a Hewlett Packard 5890 Series II GC oven (Hewlett Packard, Palo Alto, CA, USA) with a deionised water mobile phase (0.6 mL/minute). For detection, a JASCO RI-2031 Intelligent Refractive Index detector was used. Analysis was completed in 25 minutes. All JASCO-based HPLC data were acquired using the Azur version 4.6.0.0 software package (DATALYS, Saint-Martin-d'Hères, France).

### Atmospheric pressure chemical ionisation mass spectrometry

We anticipated that, when using the APCI-MS instrument, some inhibitor and fermentation samples were likely to contain high levels of analytes, which could cause competition for ionisation within the APCI source (potentially leading to signal suppression). To minimise the chances of this happening, samples were diluted prior to analysis, thereby reducing the analyte headspace concentrations. The inhibitor compound samples were diluted 10-fold, whereas those containing ethanol were diluted 250-fold. For the analysis of ethanol, the headspace sampling flow rate was also reduced. This reflected the anticipated differences in analyte concentrations, with inhibitor concentrations in the range of 0.125 to 2 g/L and ethanol concentrations in the range of 6.25 to 100 g/L.

Prior to APCI-MS analysis, aliquots (20 μL) of the ethanol calibration standards (6.25 to 100 g/L) and the fermentation samples were diluted in water (5 mL) in 28-mL flasks and sealed with 4-mm removable plugs in the caps. Similarly, 500 μL of the standard solution of inhibitor compounds (furfural, acetic acid and formic acid at 0.125 to 2 g/L) or 500 μL of pretreatment hydrolysate samples were diluted in 4.5 mL of 20 millimolar potassium phosphate buffer. Buffer pH was adjusted to a range of values between pH 1.1 and pH 3.5 by the dropwise addition of 50% vol/vol phosphoric acid.

The mass spectrometer used for headspace analysis was a modified Fisons VG Platform II single quadrupole instrument (Fisons Instruments) fitted with a custom-built APCI direct sampling interface [11]. Initially, full-scan analysis was performed over the mass to charge ratio (*m/z*) 20:250, with the analyser operating at a range of cone voltages scanned sequentially (12, 15, 18, 21, 24, 30 and 40 V). Thereafter data were collected in selected ion mode (dwell time 0.5 seconds) at the optimum cone voltage determined from the full-scan spectra. The compounds were analysed at the following cone voltages and *m/z *ratios: acetic acid, +24 V, *m/z *61; formic acid, -20 V, *m/z *45; furfural, +24 V, *m/z *97; and ethanol, +15 V, *m/z *47. The corona discharge was set to +4 kV in positive ion mode and -4 kV in negative ion mode. Equilibrated headspaces were sampled for 20 seconds with a sampling flow rate of 15 mL/minute for each compound, except ethanol, which was analysed using a sampling flow rate of 3.5 mL/minute.

### Data analysis

Microsoft Excel software (Microsoft Corp., Redmond, WA, USA) was used to fit linear regressions to the calibration and validation data. The Data Analysis ToolPak add-in (Microsoft Corp., Redmond, WA, USA) was used to perform two-way analysis of variance (ANOVA) statistical testing.

## Results and discussion

APCI-MS is a sensitive and rapid technique for the detection of compounds in the gas phase. It is capable of detecting compounds in the gas phase in the low parts per billion to low parts per million concentration range (around 0.02 to 20 mg/m^3^) [12], whilst the response rate of the instrument makes it suitable for rapid headspace profiling of samples, requiring only 20 seconds for the analysis of each sample. As such, the APCI-MS technique can analyse up to 120 samples/hour (based on a 20-second headspace sampling procedure followed by a 10-second interval between analyses whilst the selected ion response returns to baseline). Additionally, the APCI-MS technique does not require expensive consumables for sample preparation, such as syringe filters and chromatography vials, instead requiring only dilution of an aliquot of the test solution in a reusable 28-mL flask. This lower per-sample cost, however, has to be offset against the higher initial capital cost of the instrument in comparison to a typical HPLC system.

### Establishment of optimal APCI-MS operating conditions

APCI-MS headspace analysis of acetic acid, formic acid, furfural and ethanol standards was performed to determine the optimal operating parameters for detection of each analyte. Acetic acid, furfural and ethanol were all analysed in positive ion mode as the protonated molecular ion (M + 1); *m/z *61, 97 and 47, respectively. Formic acid was analysed in negative ion mode as the deprotonated molecular ion (M - 1) at *m/z *45. After selecting the appropriate ion detection mode for each compound, the cone voltage settings were adjusted to maximise signal detection. The cone voltage setting is the main parameter affecting ion transmission and fragmentation, with higher cone voltages typically resulting in greater fragmentation. Full-scan spectra were recorded at cone voltages of 12, 15, 18, 21, 24, 30 and 40 V in both positive and negative ion mode, and the optimum cone voltage was selected based upon maximising the ion response. Cone voltages selected for analysis were +15 V for ethanol, -20 V for formic acid and +24 V for both acetic acid and furfural. In subsequent experiments, the main ions of interest (as noted above) were monitored in selected ion mode at their optimum cone voltages.

### Effect of pH on equilibrium headspace partitioning of analytes

**Figure 1:**
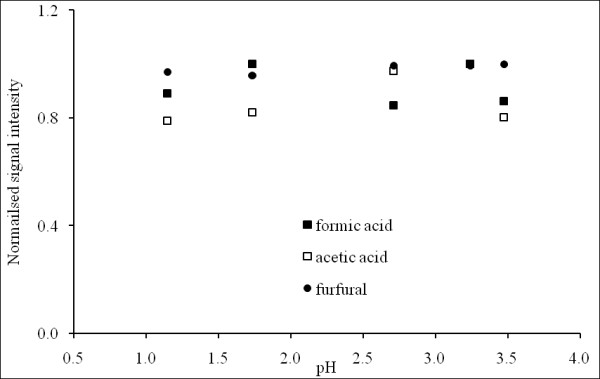
**Normalised average headspace signal intensities for formic acid, acetic acid and furfural as a function of pH (in phosphate buffer)**. Data are normalised to the maximum signal intensity observed for each compound. Each value is the mean of three replicate measurements, and from this the percentage coefficient of variance (%CV = (standard deviation ÷ mean) × 100) for each compound was calculated as formic acid (17.8%), acetic acid (12.5%) and furfural (4.4%).

The pH of the aqueous phase could potentially influence the partitioning of weak acids into the headspace, depending on the extent of dissociation of the acid. Therefore, to assess the influence of pH on volatile partitioning and its subsequent impact on the quantitative behaviour of the APCI-MS technique, headspace signal intensities of acetic acid, formic acid and furfural were each measured (after a one-hour equilibration period) in phosphate buffer in the range pH 1.1 to pH 3.5. The resultant data (Figure 1) in conjunction with ANOVA (*P *= 0.345) showed that pH had a negligible effect on the volatile partitioning of the investigated analytes across the pH range investigated. Consequently, the two weak acids, acetic acid and formic acid, must have remained largely associated over the pH range investigated. The pH values of all of the solutions were below the acid dissociation constant of acetic acid and formic acid (4.76 and 3.77, respectively), which is consistent with the partitioning behaviour observed. When strong acid or alkali is used for pretreatment, it is recommended that the pH of the hydrolysate sample be checked to ensure that it is suitable for analysis using the headspace technique. pH in the range from 1.1 to 3.5 had no major effect on the headspace signal intensities of the investigated analytes; therefore, a pH 2.5 potassium phosphate buffer was used for all subsequent experiments.

### Impact of headspace equilibration time

**Figure 2:**
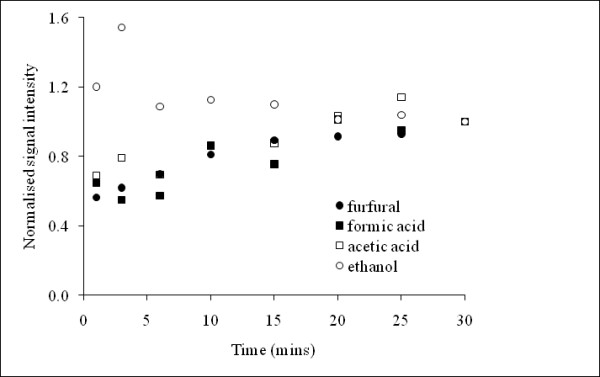
**Average headspace equilibration profiles of furfural, acetic acid, formic acid and ethanol**. The headspace signal intensity was normalised to the signal at 30 minutes. Each data point is the average of two replicate analyses. %CV values were ethanol (15.4%), formic acid (16.3%), acetic acid (17.5%) and furfural (4.7%).

Previous analyses were performed with solutions that were allowed to equilibrate for one hour prior to analysis. In this experiment, the time required for each analyte to reach headspace equilibrium was assessed by measuring the headspace signal intensity above samples over a 30-minute period. The aim was to identify the shortest equilibration time required to provide stable signal detection. We desired to minimise equilibration time to achieve high sample throughput and reduce unnecessary delays in sample assessment. Figure 2 indicates that initially the signal intensities for the three inhibitors, acetic acid, formic acid and furfural, were approximately 50% of the respective signal intensities that were eventually reached after 30 minutes. However, after 20 minutes, the headspace signals started to plateau as samples reached equilibration. The signal intensity for ethanol decreased over time, again reaching steady-state values at 20 to 30 minutes. A possible explanation for this difference in behaviour could be the surface active nature of ethanol. This hypothesis is based on the recognition that ethanol preferentially adsorbs at the air-liquid interface to form a monolayer, which can subsequently lower the liquid surface tension and increase the evaporation rate of ethanol into the headspace [13]. Leaving samples at room temperature for 20 to 30 minutes prior to analysis was found to be sufficient for equilibration between the sample and its headspace. Although, this 20-minute equilibration period may appear slow compared to rapid headspace GC techniques, it should be noted that no input from the analyst is required during the equilibration period, which can thus be routinely integrated with the analysis of large numbers of samples. Hence whilst the analyst continues to weigh out and dilute further samples, others are reaching equilibration and are then ready to be analysed. This technique is therefore considered rapid when both the minimal sample preparation steps and the APCI-MS headspace sampling time (30 seconds/sample) are taken into consideration.

### Linearity of calibration

To assess the linearity of response for each analyte, calibration series were prepared containing relevant ranges of analyte concentrations. These were selected to encompass the concentrations typically generated by our pretreatment and fermentation processes. Calibration was performed to understand the upper and lower limits of detection of the system and to determine the linearity of response of APCI-MS across a range of concentrations. All analytes were diluted in a potassium phosphate buffer (pH 2.5), except for ethanol, which was diluted in water.

**Figure 3:**
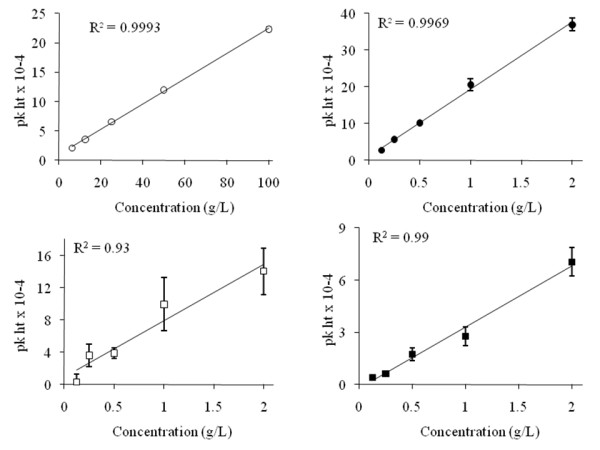
**Calibration curves for standard solutions of ethanol (white circle), furfural (black circle), acetic acid (white square) and formic acid (black square) analysed by using the atmospheric pressure chemical ionisation mass spectrometry (APCI-MS) headspace technique**. The standard solution concentration shown is that prior to dilution. Each data point is the average of three replicates. The error bars show the standard error.

Calibration data for each analyte showed a linear signal response to increasing analyte concentration over the range investigated (*R*^2 ^≥ 0.9) (Figure 3). All analytes except for acetic acid exhibited good repeatability of analysis, as was evident from the low associated standard errors (Figure 3). Acetic acid, however, showed considerable signal intensity variations at sample concentrations greater than 0.5 g/L, although the calibration series still yielded a linear regression with a correlation coefficient of 0.93.

Signal-to-noise ratios were determined for each compound at the lowest calibrant concentration (ethanol, 6.25 g/L; acetic acid, formic acid and furfural, 0.125 g/L) by comparing the signal peak height to the background noise. All signal-to-noise ratio data were greater than 3:1 (the low detection limit): acetic acid (7:1), formic acid (15:1), furfural (360:1) and ethanol (84:1). For the three inhibitors (acetic acid, formic acid and furfural), limits of detection were approximately 10-fold lower than the concentrations reported to influence bioethanol fermentation and those typical of pretreatment hydrolysates [2].

Ethanol could be readily detected in solutions derived from dilution of a 6.25 g/L standard. However, there is little interest in such low concentrations of ethanol for bioethanol production, and in this case the upper detection limit is of greater interest. Figure 3 shows that, following dilution, ethanol could be accurately analysed by APCI-MS in original sample concentrations up to 100 g/L, which is a higher concentration than that which applies to other rapid ethanol quantification techniques, such as full-equilibrium headspace GC (reported upper detection limit 12 g/L [8]). Using APCI-MS to detect ethanol at concentrations up to 100 g/L includes a range of concentrations which are more relevant to real-life bioethanol fermentation, where it is anticipated that high ethanol concentrations (a minimum of 100 g/L) are required for economic viability. Similarly, furfural concentrations up to 2 g/L could be analysed linearly using the APCI-MS headspace technique. This range is better suited to the analysis of pretreatment hydrolysate samples previously shown to contain up to 0.5 g/L furfural in comparison to the 0.125 g/L detection limits reported using full-equilibrium headspace GC [9].

All calibrants were detected and showed a satisfactory linearity of response across the range of concentrations tested (*R*^2 ^> 0.9). Acetic acid, formic acid and furfural gave a linear response up to 2 g/L, whilst for ethanol the linear range extended up to 100 g/L. APCI-MS was therefore capable of detecting each analyte over a range of concentrations that were representative of pretreatment hydrolysate and bioethanol fermentation.

### Validation

**Figure 4:**
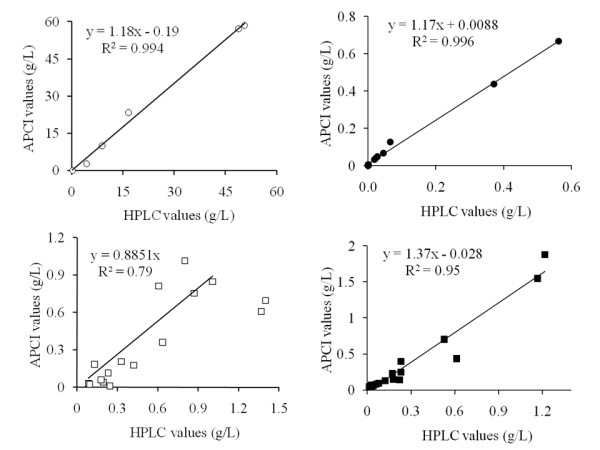
**Correlations observed between results from high-pressure liquid chromatography analysis and the APCI-MS headspace technique when analysing a fermentation time series for ethanol (white circle) and a range of pretreated wheat straw hydrolysates for furfural (black circle), acetic acid (white square) and formic acid (black square)**. Each data point represents the analysis of one sample.

Validation of the rapid APCI-MS headspace technique was achieved by comparing pretreated wheat straw hydrolysate and bioethanol fermentation data with conventional HPLC data obtained from the same samples. The strong correlations of the results derived by using these two analytical techniques (Figure 4) confirm the validity of monitoring the selected ions using the optimised APCI-MS conditions and that these correlations are representative of the analytes investigated without interference from other compounds. Since the APCI-MS technique does not involve any chromatographic separation of analytes, it was necessary to establish in this way that the selected ions monitored were truly representative of the relevant compounds. To analyse different biomass systems, it would be necessary to check the analytical validity of the application once again using a methodology similar to the one outlined in this paper.

Pretreated wheat straw hydrolysates from the designed experiment generated samples containing degradation products at varying concentrations that were dependent on the severity of the pretreatment applied. When regressing APCI-MS and HPLC data against one another, strong correlations were observed between the two analytical techniques in detecting ethanol and the inhibitors furfural and formic acid (*R*^2 ^> 0.95) (Figure 4). Acetic acid, however, showed a lower degree of correlation between the two techniques, with an *R*^2 ^value of 0.79. Acetic acid detection using the APCI-MS headspace technique was in general more variable (Figures 3 and 4). Signal intensities for acetic acid detected using the APCI-MS technique exhibited a loss in quantitative behaviour, which is suspected to have been caused by the presence of other analytes competing for proton transfer during ionisation. Acetic acid has a proton affinity of close to 748 kJ/mol [12], whereas certain volatile organic compounds present in biomass hydrolysates can have significantly higher proton affinities and hence inhibit the quantitative ionisation of acetic acid. For example, furfural and HMF have proton affinities of the order of 820 kJ/mol [14]. In comparison, formic acid, which is chemically similar to acetic acid, did not exhibit this behaviour, owing to the detection of this analyte in negative ion mode. This effect can be seen in Figure 4, where pretreated wheat straw hydrolysate samples which contained acetic acid at 1.4 g/L according to HPLC analysis gave a value of only 0.7 g/L when analysed by APCI-MS. The APCI-MS clearly underestimated the amount of acetic acid present in this instance. Removal of the two data points contributing to this difference from the linear regression line was necessary to increase the *R*^2 ^value from 0.6 to 0.8. Detection of high levels of acetic acid in pretreated biomass samples by APCI-MS was affected by a loss in quantitative behaviour. This could potentially be alleviated by further diluting the samples beyond the 10-fold dilution employed by using the method described herein. In this instance, the technique that we used was therefore only quantitative for studying levels of acetic acid, which have been shown to stress yeast strains [15], but not at concentrations shown to be inhibitory (1.6 g/L).

Analysis of samples from a bioethanol fermentation time course provided a range of ethanol concentrations for analysis by HPLC and APCI-MS. APCI-MS analysis indicated that ethanol levels were within the range encompassed by the calibration curve and showed good agreement with HPLC data for the same samples (*R*^2 ^= 0.994) (Figure 4). APCI-MS headspace analysis was therefore found to offer a rapid analytical technique for quantifying ethanol concentrations during fermentation. Although the present bioethanol fermentation was performed using a synthetic medium, the impact of using an actual lignocellulosic hydrolysate or other complex fermentation medium on the quantification of ethanol in the headspace would be anticipated to be minimal. This is in part due to the fact that ethanol is present at concentrations which substantially exceed those of other volatile fermentation products; hence slight changes in concentrations of the latter would not affect the quantitative ionisation of ethanol in the APCI source. In addition, the precise nature of the medium would be anticipated to have a minor impact on the array of volatile chemicals generated through fermentation, compared, for example, with the strain of organism used to conduct the fermentation.

It should be noted that the capability of the APCI-MS technique to selectively quantify analytes in the gas phase only permits the quantification of volatile pretreatment hydrolysate degradation products (and fermentation ethanol). A limitation in the application of this technique for screening degradation products from lignocellulosic pretreatment hydrolysates is that it cannot extensively quantify all currently recognised lignocellulosic pretreatment degradation products, such as levulinic acid and *p*-coumaric acid, which are nonvolatile. Therefore, by using the APCI-MS technique, only a selective range of pretreatment degradation products can be quantified, and these may not wholly reflect the extent of hydrolysate toxicity towards fermentation.

## Conclusions

APCI-MS headspace analysis is a rapid analytical technique capable of quantifying three key pretreatment degradation products, acetic acid, formic acid and furfural, as well as fermentation ethanol, with high selectivity and with minimal sample preparation. Samples typically required dilution followed by a 20-minute equilibration period and 20 seconds for headspace analysis, as compared to HPLC-based methods, which require lengthy chromatographic run times. The gas phase selectivity of the APCI-MS technique enables the rapid quantification of volatile or semivolatile fermentation-inhibitory compounds within complex hydrolysates and fermentation media. However, nonvolatile inhibitor compounds cannot likewise be analysed. In conclusion, APCI-MS is proposed to offer a cost-effective and rapid approach to the detection of three inhibitory compounds and ethanol during the conversion of lignocellulosics to bioethanol.

## Competing interests

The authors declare that they have no competing interests.

## Authors' contributions

RSL coordinated the experimental design and helped draft the manuscript. SJW carried out the calibrant preparation, HPLC and APCI-MS equipment setup and drafted the Methods section. SMD helped SJW run the samples, prepare the pretreatment samples and draft the manuscript. DJC and KAS are the academics responsible for funding and supervising the research in addition to proofreading the manuscript and providing feedback. All authors read and approved the final manuscript.

## References

[B1] Wyman CE Dale BE Elander RT Holtzapple M Ladisch MR Lee YY Coordinated development of leading biomass pretreatment technologies Bioresour Technol 2005 96 1959 1966 10.1016/j.biortech.2005.01.010 16112483

[B2] Almeida JR Modig T Petersson A Hähn-Hägerdal B Lidén G Gorwa-Grauslund MF Increased tolerance and conversion of inhibitors in lignocellulosic hydrolysates by *Saccharomyces cerevisiae* J Chem Technol Biotechnol 2007 82 340 349 10.1002/jctb.1676

[B3] Palmqvist E Hähn-Hägerdal B Fermentation of lignocellulosic hydrolysates. II: inhibitors and mechanisms of inhibition Bioresour Technol 2000 74 25 33 10.1016/S0960-8524(99)00161-3

[B4] Dogaris I Karapati S Mamma D Kalogeris E Kekos D Hydrothermal processing and enzymatic hydrolysis of sorghum bagasse for fermentable carbohydrates production Bioresour Technol 2009 100 6543 6549 10.1016/j.biortech.2009.07.046 19692234

[B5] Kabel MA Bos G Zeevalking J Voragen AGJ Schols HA Effect of pretreatment severity on xylan solubility and enzymatic breakdown of the remaining cellulose from wheat straw Bioresour Technol 2007 98 2034 2042 10.1016/j.biortech.2006.08.006 17029957

[B6] Lee JM Shi J Venditti RA Jameel H Autohydrolysis pretreatment of Coastal Bermuda grass for increased enzyme hydrolysis Bioresour Technol 2009 100 6434 6441 10.1016/j.biortech.2008.12.068 19665372

[B7] Decker SR Brunecky R Tucker MP Himmel ME Selig MJ High-throughput screening techniques for biomass conversion Bioenerg Res 2009 2 179 192 10.1007/s12155-009-9051-0

[B8] Li H Chai XS Deng Y Zhan H Fu S Rapid determination of ethanol in fermentation liquor by full evaporation headspace gas chromatography J Chromatogr A 2009 1216 169 172 10.1016/j.chroma.2008.11.024 19041976

[B9] Li H Chai XS Zhan H Fu S Rapid determination of furfural in biomass hydrolysate by full evaporation headspace gas chromatography J Chromatogr A 2010 1217 7616 7619 10.1016/j.chroma.2010.09.073 20970806

[B10] Ashraf N Linforth RST Bealin-Kelly F Smart K Taylor AJ Rapid analysis of selected beer volatiles by atmospheric pressure chemical ionisation-mass spectrometry Int J Mass Spectrom 2010 294 47 53 10.1016/j.ijms.2010.05.007

[B11] Linforth RST Taylor AJ Apparatus and Methods for the Analysis of Trace Constituents in Gases 1999 European Patent EP 0819937 A2

[B12] Taylor AJ Linforth RST Harvey BA Blake A Atmospheric pressure chemical ionisation mass spectrometry for in vivo analysis of volatile flavour release Food Chem 2000 71 327 338 10.1016/S0308-8146(00)00182-5

[B13] Tsachaki M Linforth RST Taylor AJ Dynamic headspace analysis of the release of volatile organic compounds from ethanolic systems by direct APCI-MS J Agric Food Chem 2005 53 8328 8333 10.1021/jf051202n 16218684

[B14] Jafari MT Khayamian T Simultaneous determination of 2-furfural and 5-methyl-2-furfural using corona discharge ion mobility spectrometry Anal Sci 2009 25 801 805 10.2116/analsci.25.801 19531891

[B15] Narendranath NV Thomas KC Ingledew WM Effects of acetic acid and lactic acid on the growth of *Saccharomyces cerevisiae *in a minimal medium J Ind Microbiol Biotechnol 2001 26 171 177 10.1038/sj.jim.7000090 11420658

